# Supplementary material for: Inoculation with Azospirillum brasilense Strains AbV5 and AbV6 Increases Nutrition, Chlorophyll, and Leaf Yield of Hydroponic Lettuce
Source: Plants (Basel). 2023 Aug 29;12(17):3107. doi: 10.3390/plants12173107 (PMC10490540; doi:10.3390/plants12173107)
Supplement: Supplementary file 1 [file plants-12-03107-s001.zip › plants-2496982-supplementary.pdf]

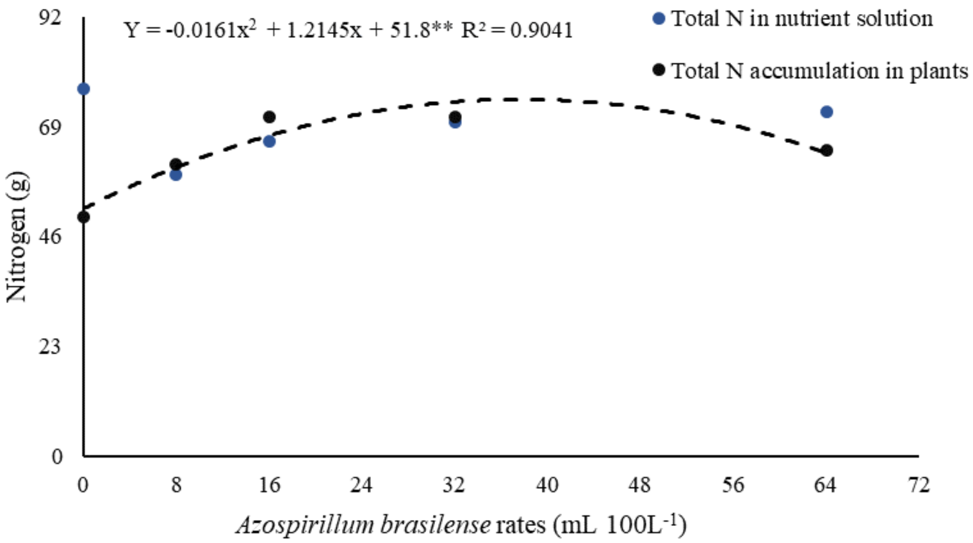

Figure S1. Total nitrogen accumulation in lettuce plants on the hydroponic cultivation bench and total nitrogen supply via nutrient solution throughout the growing period according to the doses of *Azospirillum brasilense* via nutrient solution.

Table S1. Summary of analysis of variance of shoot fresh matter (SFM), root fresh matter (RFM), shoot dry matter (SDM), root dry matter (RDM), number of leaves (NL), and yield (YLD).

Source of variation	Mean Squares					
	SFM	RFM	SDM	RDM	NL	YLD
Block	680.80	4.55	8.55	0.02	3.71	0.26
Doses	8862.80**	177.99**	7.79**	0.42**	9.61*	3.37**
Residue	302.30	13.77	0.52	0.01	2.29	0.11
CV (%)	6.76	9.51	7.37	5.94	7.94	5.76
Regression						
Linear	0.01**	0.001**	0.001**	0.001**	0.03*	0.001**
Quadratic	0.001**	0.09^{ns}	0.32^{ns}	0.001**	0.01**	0.008**
Deviation	0.47^{ns}	0.95^{ns}	0.74^{ns}	0.06^{ns}	0.32^{ns}	0.47^{ns}

** significant at 1%, * significant at 5%, ^{ns} not significant, CV - coefficient of variation.

Table S2. Summary of analysis of variance of nitrogen (N), phosphorus (P), potassium (K), calcium (Ca), magnesium (Mg), sulfur (S), boron (B), iron (Fe), manganese (Mn), and zinc (Zn) accumulation in shoots and roots of lettuce plants.

Source of variation	Mean Squares - Shoot accumulation									
	N	P	K	Ca	Mg	S	B	Fe	Mn	Zn
Block	3.05	0.22	9.54	1.22	0.11	0.03	5.08	8940	220.9	128.1
Doses	4.02**	0.44**	7.65**	1.37*	0.11**	0.03*	3.92**	13646**	513.1**	157.6**
Residue	0.71	0.02	1.23	0.26	0.01	0.007	0.35	921	53.40	18.85
CV (%)	10.24	7.65	12.20	15.44	10.41	13.29	11.08	8.34	13.33	10.07
Regression										
Linear	0.07^{ns}	0.01**	0.02*	0.63^{ns}	0.01**	0.01**	0.02*	0.76^{ns}	0.01**	0.05*
Quadratic	0.01**	0.01**	0.01**	0.02*	0.01**	0.09^{ns}	0.01**	0.01**	0.06^{ns}	0.01**
Deviation	0.12^{ns}	0.06^{ns}	0.12^{ns}	0.06^{ns}	0.07^{ns}	0.53^{ns}	0.13^{ns}	0.08^{ns}	0.43^{ns}	0.06^{ns}
Source of variation	Mean Squares - Root accumulation									
	N	P	K	Ca	Mg	S	B	Fe	Mn	Zn
Block	0.004	0.003	0.003	0.0006	0.002	0.002	0.004	72.90	0.37	1.37
Doses	0.41**	0.006**	0.20**	0.002^{ns}	0.008**	0.02**	0.53**	1469**	14.09**	12.6**
Residue	0.009	0.0009	0.004	0.004	0.0008	0.001	0.009	62.62	0.50	0.42
CV (%)	9.64	9.48	8.22	20.34	10.70	12.65	8.76	7.36	8.85	7.77
Regression										
Linear	0.01**	0.05*	0.01**	0.94^{ns}	0.02*	0.01**	0.01**	0.01**	0.01**	0.03*
Quadratic	0.01**	0.001**	0.01**	0.64^{ns}	0.01**	0.01**	0.01**	0.01**	0.01**	0.01**

Deviation	0.06^{ns}	0.80^{ns}	0.06^{ns}	0.53^{ns}	0.54^{ns}	0.38^{ns}	0.11^{ns}	0.20^{ns}	0.08^{ns}	0.60^{ns}

** significant at 1%, * significant at 5%, ^{ns} not significant, CV - coefficient of variation.

Table S3. Summary of analysis of variance of relative water content (RWC), leaf membrane integrity index (MII), internal CO_2 concentration (*Ci*), stomatal conductance (*gs*), net photosynthesis rate (*A*), transpiration (*E*), water use efficiency (*WUE*), and total chlorophyll content (ChlT).

Source of variation	Mean Squares							
	RWC	MII	*Ci*	*gs*	*A*	*E*	*WUE*	ChlT
Block	28.18	1.25	427.8	3049	1.16	0.39	0.03	0.001
Doses	293.1**	88.70**	7510**	151437**	72.24**	6.52**	0.40**	0.13**
Residue	14.52	1.33	799.6	4100	1.95	0.77	0.02	0.001
CV (%)	4.38	1.29	8.36	9.70	10.94	8.55	10.10	3.69
Regression								
Linear	0.01**	0.11^{ns}	0.01**	0.01**	0.18^{ns}	0.01**	0.01**	0.01**
Quadratic	0.01**	0.00**	0.05*	0.01**	0.01**	0.01**	0.01**	0.00**
Deviation	0.06^{ns}	0.80^{ns}	0.08^{ns}	0.08^{ns}	0.09^{ns}	0.06^{ns}	0.06^{ns}	0.07^{ns}

** significant at 1%, * significant at 5%, ^{ns} not significant, CV - coefficient of variation.

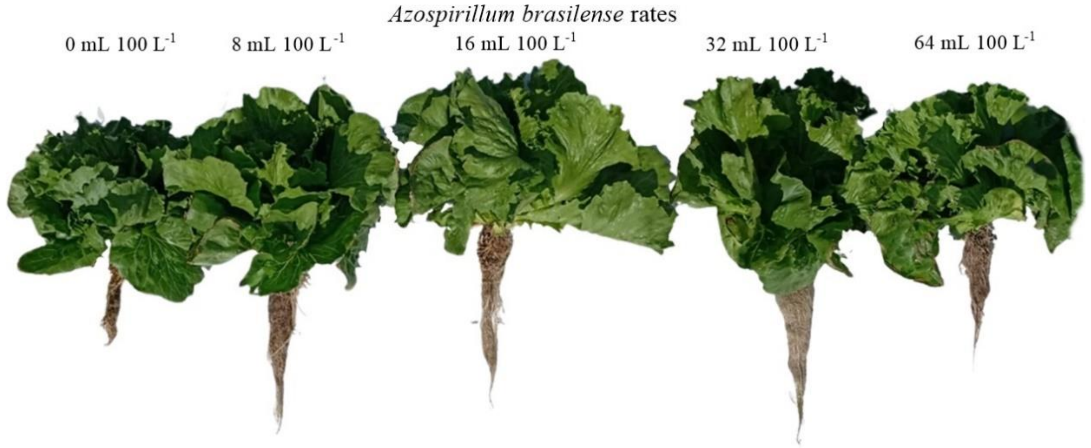

Fig. S2. *Azospirillum brasilense* doses increase shoot and root growth of iceberg lettuce plants in hydroponics.

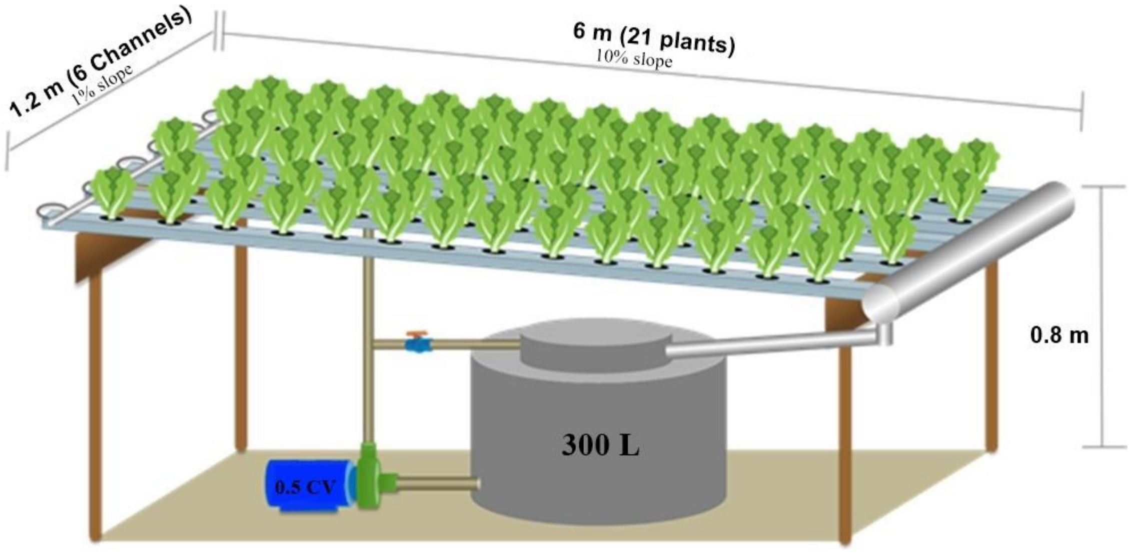

Fig. S3. Schematic of the hydroponics bench used in the test conduction. Ilha Solteira, SP, 2021.
